# Supplementary material for: Electromagnetic reprogrammable coding-metasurface holograms
Source: Nat Commun. 2017 Aug 4;8:197. doi: 10.1038/s41467-017-00164-9 (PMC5543116; doi:10.1038/s41467-017-00164-9)
Supplement: Supplementary file 1 — Supplementary Information [file 41467_2017_164_MOESM1_ESM.pdf]

File name: Supplementary Information
Description: Supplementary figures and supplementary notes.

File name: Supplementary Movie 1
Description: The evolution of the holographic images with increasing observation distance with and without adaptive tuning.

File name: Supplementary Movie 2
Description: Illustration of the image quality with the progress of iterations.

File name: Peer review file
Description:

Supplementary Note 1: Unit cell of the 1-bit programmable metasurface

Supplementary Figure 1 illustrates the schematic of the unit cell in the 1-bit programmable coding metasurface and the equivalent circuit model for the diode.

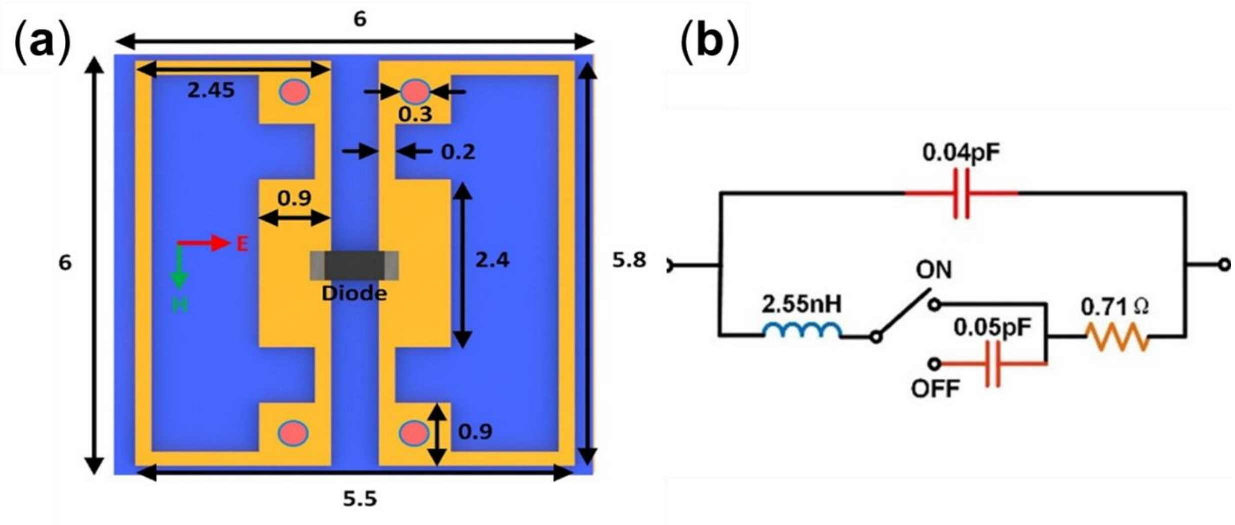

Supplementary Figure 1 The unit cell (meta-atom) of the 1-bit programmable coding metasurface hologram and the equivalent circuit models of the diode. (**a**) Top view of the unit cell with detailed geometrical parameters (unit: mm). (**b**) The equivalent circuit models of the diode biased at the 'ON' and 'OFF' states.

Supplementary Note 2: Forward propagation model of the 1-bit programmable metasurface

Here, the effective current inversion technique is employed to find the induced current source $\mathbf{J}^{(S)}$ within the macro metasurface particles illuminating by a linearly polarized plane wave, where the superscript $S = 1$ (0) corresponds to the macro particle at the '1' ('0') state. For a metasurface hologram with $N_x \times N_y$ controllable particles illuminating by the plane wave, the resultant co-polarized radiation in the image plane is approximately written as

$$E(\mathbf{r}) = \sum_{n_x=1}^{N_x}\sum_{n_y=1}^{N_y}\int_\Delta g(\mathbf{r}, \mathbf{r}_{n_x,n_y} + \delta\mathbf{r}) J^{(S_{n_x,n_y})}(\delta\mathbf{r}) d\delta\mathbf{r} \quad (1)$$

where $g\left(\mathbf{r}, \mathbf{r}_{n_x,n_y}\right) = \frac{exp\left(jk_0\left|\mathbf{r}-\mathbf{r}_{n_x,n_y}\right|\right)}{4\pi\left|\mathbf{r}-\mathbf{r}_{n_x,n_y}\right|}$ is the three-dimensional Green's function in free space, k_0 is the operational wavenumber, and $\mathbf{r}_{n_x,n_y}$ denotes the central coordinate of the $(n_x, n_y)^{th}$ macro metasurface particle. In Supplementary Eq. (1), the integration is implemented over the surface of the macro particle denoted by Δ, and the double summation is performed over all pixels of the coding metasurface. S_{n_x,n_y} represents the state of the (n_x, n_y)th pixel, where n_x and n_y denote the running indices of the pixel of the 1-bit coding metasurface along the x and y directions, respectively. Note that the image E is linearly related to the current J over the reprogrammable metasurface hologram. Under the far-field approximation, the spatial bandwidth of E is limited by the maximum value of $|\mathbf{k}_{n_x,n_y} + \mathbf{k}_\mathrm{d}|$, and is determined by the maximum size of the coding metasurface, where $\mathbf{k}_{n_x,n_y} \approx k_0\mathbf{r}_{n_x,n_y}/r_{n_x,n_y}$ and $\mathbf{k}_\mathrm{d} \approx k_0\mathbf{r}/r$. In this way, it can be deduced that the achievable resolution of the holographic image is of the order of $O(\lambda R/D)$, where λ is the operating wavelength, R is the observation distance, and D is the maximum size of the reprogrammable metasurface hologram.

Supplementary Note 3: Observation-distance adaptive feature

As pointed out in the main text, the hologram optimized by the modified GS algorithm relies strongly on the observation distance (Z_r) of the image plane. In **Supplementary Figure 2a**, we demonstrated the holographic images of the letter "S" at different observation distances of 400mm, 440mm, 480mm, and 700mm, where the hologram is designed at Z_r =400mm. The corresponding holographic results are shown in **Supplementary Figure 2b**, where the hologram is adaptively reprogrammed with the change of the observation distance. Apparently, the hologram needs to be tuned to get clearer holographic images when the image plane is relocated; consequently, the quality of holography image could be remarkably improved by adjusting the hologram. As illustrated in

Supplementary Figure 2b, the dynamic hologram could work in a relatively wide observation range, e.g., from 400mm to 700mm, when the hologram is adaptively controlled. A quantitative analysis is presented in **Figure 5** in the main text.

Supplementary Figure 2 (**a**) the image of a letter "S" at different observation distances (Z_r), where the hologram is designed at Z_r=400mm; (**b**) the image of a letter "S" at different observation distances, where the hologram is adaptively tuned with the change of the Z_r.

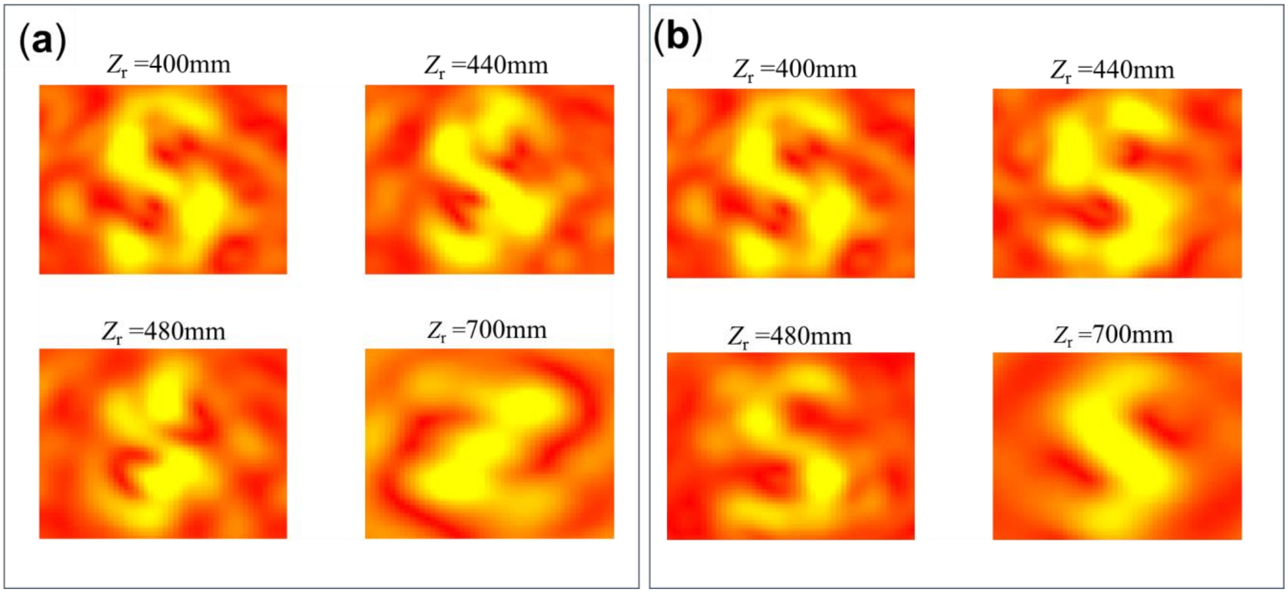

Supplementary Note 4: Frequency adaptive feature

Although the reprogrammable metasurface is designed at 7.8 GHz, the hologram works well within a working frequency band of 0.5GHz. **Supplementary Figure 3** gives the quantitative analysis of the image quality when the hologram is programmed to be tuned or not with the change the operational frequency. Additionally, **Supplementary Figure 4** provides the images of a letter "P" at different sampling frequencies, where the holographic images are obtained by adjusting the metasurface hologram accordingly while changing the working frequencies. Once again, the frequency adaptive feature can also bring us improvements on the holographic image quality compared with the fixed hologram.

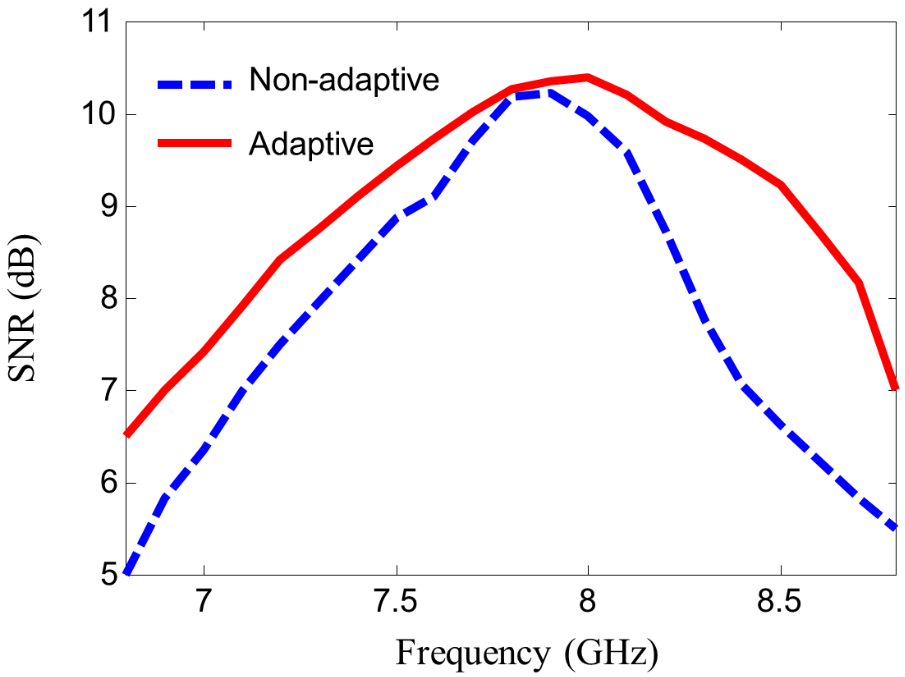

Supplementary Figure 3 | The dependence of the SNR of the holographic images as the function of the frequency. The red line represents the SNR of the holographic image by adaptively tuning the metasurface hologram with the change of the working frequencies; however, the blue line denotes the SNR of the holographic images with the hologram designed with the operational frequency of 7.8 GHz.

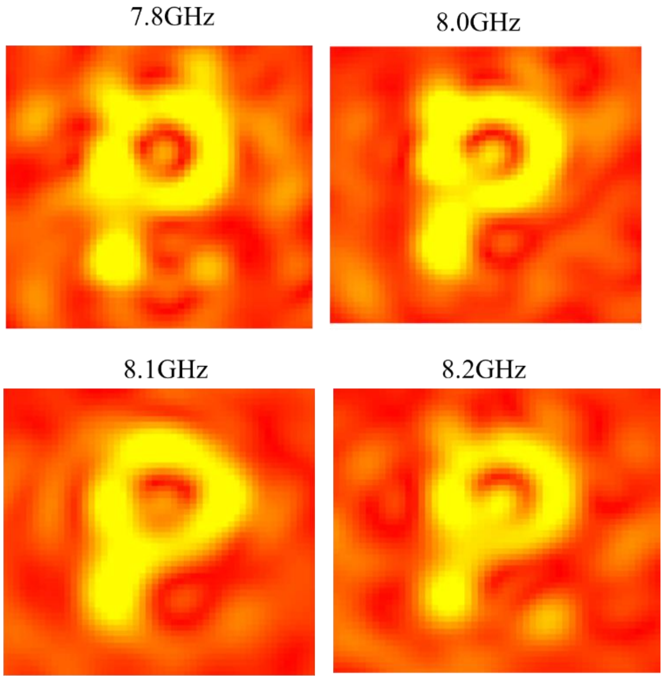

Supplementary Figure 4 | The images of letter "P" at different sampling frequencies, in which the holographic images are obtained by adjusting the metasurface holograms accordingly while changing the working frequencies.

Supplementary Note 5: The hologram efficiency

As discussed in main text, there is some discrepancy of efficiency between the measurement and theory. We think that besides the reasons of imperfect quality of commercial pin-diode product, there are two major possible factors to cause this relatively low conversion efficiency (~60%).

First, there is the quantization loss due to the phase level, as illustrated in **Supplementary Figure 5**. We observe that the choice of two quantization levels will give the maximum attainable efficiency to nearly 70% of the optimal design efficiency.

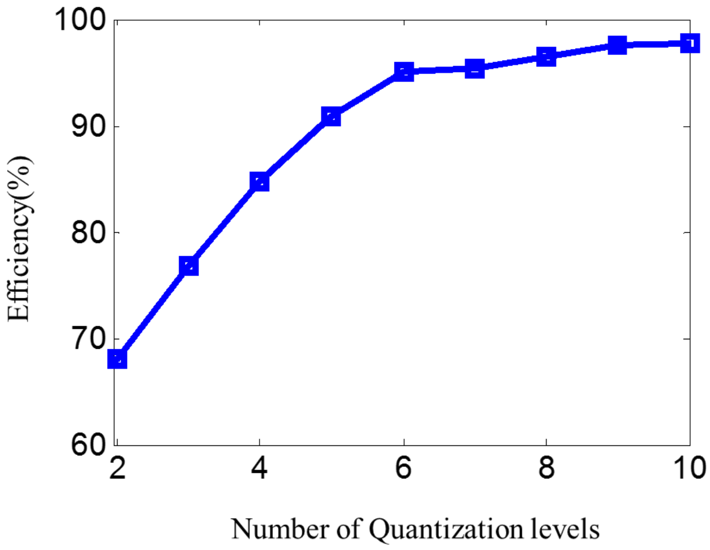

Supplementary Figure 5 The quantization efficiency penalty in relative to the continuous phase solution, as a function of the number of quantization levels of the phase.

Secondly, the metasurface with finite extension is illuminated by a horn antenna in our experiments, therefore there exists finite divergence angle in the incident beam. However, the simulation results are obtained when the metasurface atom is illuminated by an idea plane wave in the periodic boundary condition. Assuming that f is the field at the hologram generated by an ideal plane-wave illumination, and g represents the actual illumination beam profile at the hologram plane. Thus the real holographic image denoted by h can be approximated as the two-dimensional convolution of f and $\mathcal{F}\{g\}$, i.e., $h \approx f \otimes \mathcal{F}\{g\}$, where $\mathcal{F}\{g\}$ denotes the two-dimensional Fourier

transformation of g. Apparently, the convolution kernel $\mathcal{F}\{g\}$ will lead to a blurred holographic image, which is responsible for the efficiency reduction.

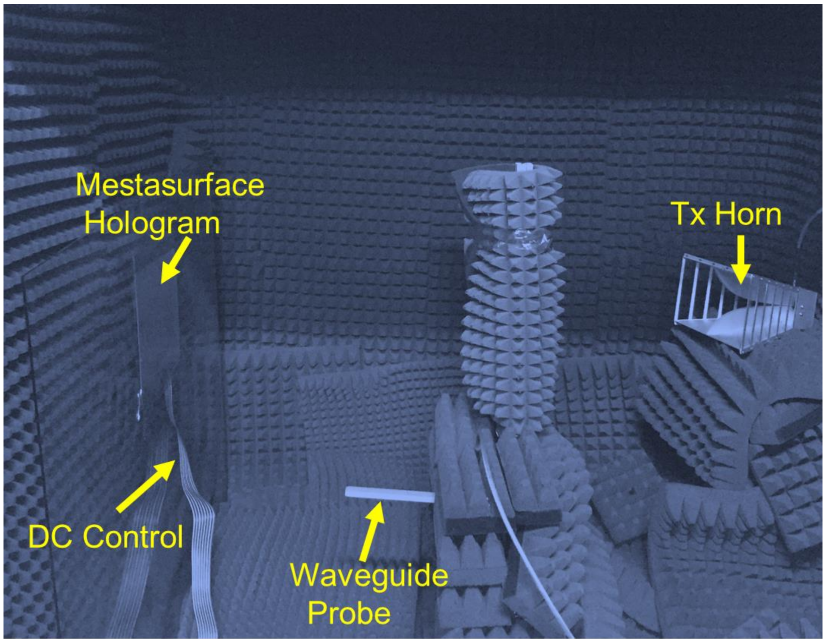

Supplementary Figure 6 | The photograph of proof-of-concept experimental setup, which is composed of a transmitting (Tx) horn antenna, a receiving (Rx) waveguide probe, and an Agilent vector network analyzer (Agilent E5071C).
